# Optoelectronic Properties and the Electrical Stability of Ga-Doped ZnO Thin Films Prepared via Radio Frequency Sputtering

**DOI:** 10.3390/ma9120987

**Published:** 2016-12-06

**Authors:** Shien-Uang Jen, Hui Sun, Hai-Pang Chiang, Sheng-Chi Chen, Jian-Yu Chen, Xin Wang

**Affiliations:** 1 Institute of Physics, Academia Sinica, Taipei 115, Taiwan; physjen@gate.sinica.edu.tw; 2 Institute of Optoelectronic Science, National Taiwan Ocean University, Keelung 202, Taiwan; hpchiang@ntou.edu.tw (H.-P.C.); alexunh@126.com (J.-Y.C.); 3 Institute of Materials Science and Engineering, Ocean University of China, 238 Songling Road, Qingdao 266100, China; sunhuichn@hotmail.com (H.S.); wangxinhd@ouc.edu.cn (X.W.); 4 Department of Materials Engineering and Center for Thin Film Technologies and Applications, Ming Chi University of Technology, Taipei 243, Taiwan; 5 Department of Electronic Engineering, Chang Gung University, Taoyuan 333, Taiwan

**Keywords:** Ga-doped ZnO (GZO) thin films, optoelectronic properties, rf sputtering, electrical stability

## Abstract

In this work, Ga-doped ZnO (GZO) thin films were deposited via radio frequency sputtering at room temperature. The influence of the Ga content on the film’s optoelectronic properties as well as the film’s electrical stability were investigated. The results showed that the film’s crystallinity degraded with increasing Ga content. The film’s conductivity was first enhanced due to the replacement of Zn^2+^ by Ga^3+^ before decreasing due to the separation of neutralized gallium atoms from the ZnO lattice. When the Ga content increased to 15.52 at %, the film’s conductivity improved again. Furthermore, all films presented an average transmittance exceeding 80% in the visible region. Regarding the film’s electrical stability, GZO thermally treated below 200 °C exhibited no significant deterioration in electrical properties, but such treatment over 200 °C greatly reduced the film’s conductivity. In normal atmospheric conditions, the conductivity of GZO films remained very stable at ambient temperature for more than 240 days.

## 1. Introduction

Transparent conductive oxides (TCOs) have been widely studied in the last few decades due to their high electrical conductivity and good optical transmittance in the visible light region. They can be used in fields such as solar cells, flat-panel displays, light emitting diodes, and smart windows [1,2,3,4,5]. In particular, Sn-doped In_2_O_3_ (ITO) films present outstanding optoelectronic performance and have been widely used in various commercial domains [6,7,8]. However, due to the toxicity and limited availability of indium, further applications of ITO are restricted, so novel alternative materials must be developed [9,10]. ZnO-based oxides are considered one of the most promising transparent conductive oxides due to their low production cost, non-toxicity, low growth temperature, and high adaptability to various substrates [11,12,13,14,15,16]. Nevertheless, their practical feasibility is strongly restricted by the poor electrical properties of intrinsic ZnO thin films. In order to overcome this drawback, many studies have attempted to improve the performance of ZnO. One approach is to enhance the electrical and optical properties of ZnO thin films by trivalent Ga doping [17,18,19,20,21].

In this work, Ga-doped ZnO (GZO) thin films were prepared via radio frequency (rf) sputtering on ZnO and Ga_2_O_3_ targets. The influences of varying the sputtering power applied to the Ga_2_O_3_ target on the composition and optoelectronic properties of the films were investigated. The film’s thermal and time stabilities are also discussed.

## 2. Results and Discussion

**Figure 1:**
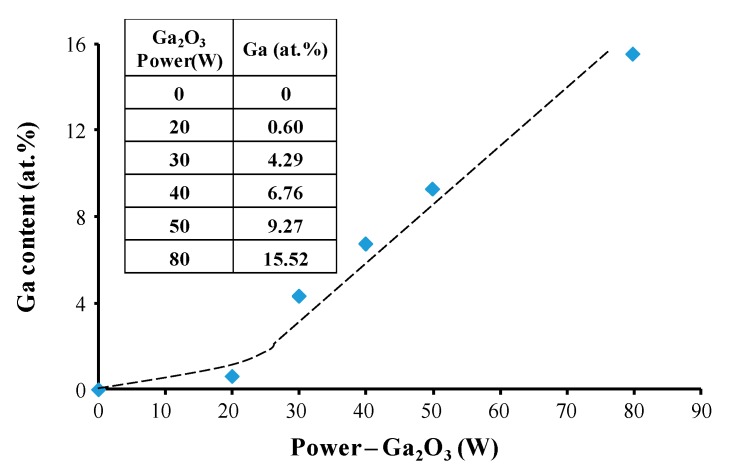
Ga content in ZnO thin films as a function of the sputtering power applied on the Ga_2_O_3_ target.

The influence of the sputtering power applied to the Ga_2_O_3_ target (*P*_Ga2O3_) on the variation in Ga content in the films was first investigated. The results are shown in Figure 1. With increases in *P*_Ga2O3_, Ga content initially increased slightly (*P*_Ga2O3_ < 20 W) before rising significantly as *P*_Ga2O3_ rose above 20 W. The actual Ga content of the films with various *P*_Ga2O3_ is given in the inset table. ZnO with 0.60 at % Ga thin film was obtained when *P*_Ga2O3_ was fixed at 20 W. When *P*_Ga2O3_ was above 30 W, Ga content increased to more than 4.29 at %. As reported by H. Gomez [22] and R. Wang [23], the solubility limit of Ga in ZnO is around 2–3 at % at room temperature. If more Ga is introduced into ZnO thin film, the redundant gallium atoms will segregate from the ZnO lattice and form neutralized gallium species in the grain boundaries of ZnO.

**Figure 2:**
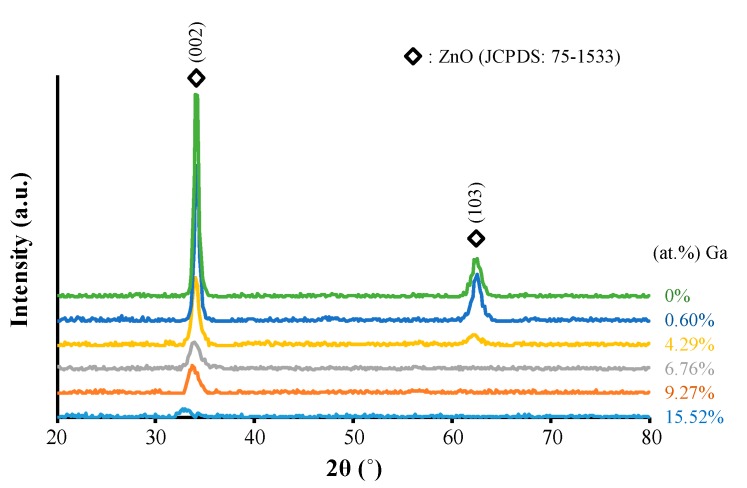
X-ray diffraction (XRD) patterns of ZnO thin films with varying amounts of Ga.

The X-ray diffraction patterns of the ZnO thin films with varying amounts of Ga are shown in Figure 2. When the doping content of Ga was about 0.6 at %, no obvious peak shift was observed. Even though the ionic radius of Ga^3+^ (0.062 nm) is smaller than that of Zn^2+^ (0.074 nm) [24], the amount of Zn^2+^ cations replaced by Ga^3+^ cations was at a very low level, so the contraction in lattice parameters was not considerable. However, when the content of Ga^3+^ cations exceeded 4.29 at %, the ZnO diffraction peaks moved to lower diffraction angles. This movement became more pronounced with the rise in Ga content owing to redundant Ga atoms entering into the ZnO lattice and forming Ga interstitial (Ga*_i_*) defects, which enlarged the lattice parameters of ZnO. Moreover, as the extrinsic defects of (Ga_Zn_) (the substitution of Zn by Ga), and (Ga*_i_*) were introduced into the ZnO films, the films’ crystallinity degraded as the Ga content increased.

**Figure 3:**
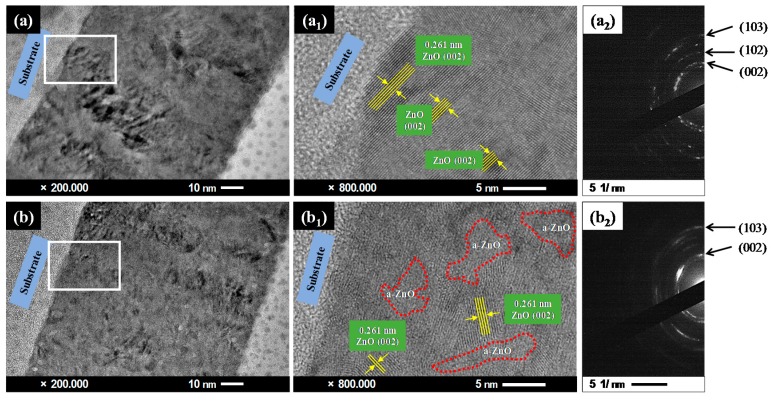
TEM observation and the corresponding high-resolution TEM images of Ga-doped ZnO (GZO) films deposited with Ga_2_O_3_ powers of 30 W (**a**,**a_1_**) and 50 W (**b**,**b_1_**), and their electron diffraction patterns: 30 W (**a_2_**) and 50 W (**b_2_**).

The degradation of the film’s crystallinity with increasing Ga content was revealed by TEM observation (Figure 3). The crystallinity of GZO films with more gallium (*P*_Ga2O3_ = 50 W) was found to be inferior to those with less gallium (*P*_Ga2O3_ = 30 W). Some amorphous domains (highlighted by red dotted lines) embedded in the crystallized ZnO are visible in Figure 3b_1_, but none can be found in Figure 3a_1_. Additionally, in comparing the selected area electron diffraction (SAED) patterns of these two films, the conversion of the diffraction spots (Figure 3a_2_) to the diffraction rings (Figure 3b_2_) indicated an obvious reduction in grain size, which degraded the film’s crystallinity.

**Figure 4:**
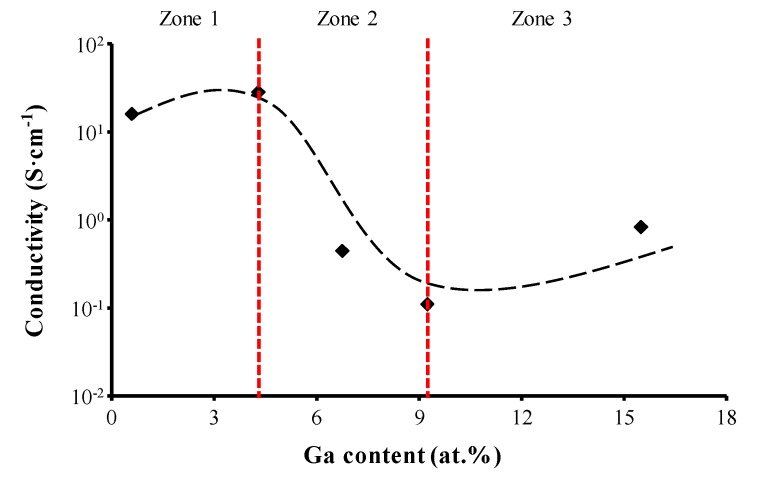
The variation in film conductivity as a function of Ga content in ZnO films.

The film’s conductivity as a function of Ga content in ZnO films is shown in Figure 4. The conductivity of the film without Ga doping was too low to be detected under our experimental conditions. After the Ga doping of ZnO, the film’s conductivity significantly improved when the doping concentration was less than 4.29 at %, a range (Zone 1) in which Zn^2+^ cations were effectively replaced by Ga^3+^ cations. However, this improvement of the film’s conductivity disappeared when the Ga^3+^ content increased to a range of 4.29–9.27 at % (Zone 2), due mainly to the Ga content exceeding its solubility in ZnO and neutralized gallium atoms separating from the ZnO lattice. These neutralized species could not contribute to the generation of free electrons and enhanced the scattering effect of the carriers in ZnO films. Therefore, the film’s conductivity declined in this range. Upon a further increase in Ga content to 15.52 at % (Zone 3), the film’s conductivity improved because the neutralized gallium atoms gathered together and formed Ga clusters due to the high Ga doping concentration. Given that the work functions of gallium and intrinsic ZnO are about 4.2 eV and 4.5 eV, respectively [25], ohmic contact occurred between the metallic gallium and n-type ZnO. As a result, the abundant electrons in gallium easily transferred into the ZnO, and the film’s conductivity thus increased.

**Figure 5:**
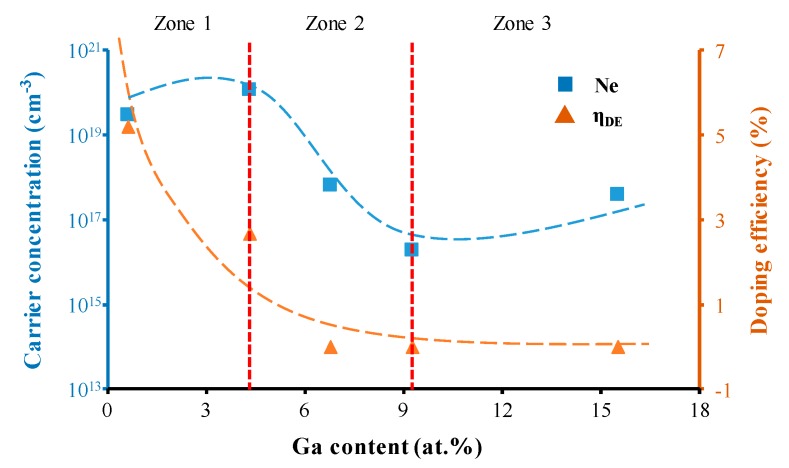
The variation in carrier concentration Ne and doping efficiency η_DE_ as a function of Ga content (the bulk ZnO density of 5.606 g/cm^3^ was used to estimate η_DE_).

The variation in the carrier concentration in the films with increasing Ga concentration was analyzed via Hall measurement, and the results are shown in Figure 5. The carrier concentration exhibited a trend similar to that of the film’s conductivity and revealed the formation and development of neutralized gallium species. When the Ga content was relatively low (Zone 1), most gallium atoms were ionized and replaced Zn^2+^ cations. Free electrons were derived from this replacement. Thus, the carrier concentration increased with rising gallium content. In Zone 2, the carrier concentration decreased due to an increasing number of gallium atoms separating from the ZnO lattice and forming neutral defects, which did not contribute to the generation of free electrons. Furthermore, the intrinsic donor defects of oxygen vacancies could be occupied by Ga atoms, which reduced the carrier concentration. In Zone 3, gallium atoms gathered into clusters, and more electrons could easily transfer from gallium clusters to ZnO films. Thus, the carrier concentration increased. 

In order to understand the effective doping amount and the electrical activity of gallium in the ZnO films, the doping efficiency (η_DE_) of Ga^3+^ cations in ZnO was also investigated (Figure 5). η_DE_ is defined as the ratio of the carrier concentration to the atomic ratio of Ga/(Ga + Zn), where the free electrons in ZnO films are mainly considered to be created by Ga substitution. As shown in Figure 5, the doping efficiency decreased with increasing gallium content, suggesting that more gallium atoms separated from the ZnO as gallium content increased.

**Figure 6:**
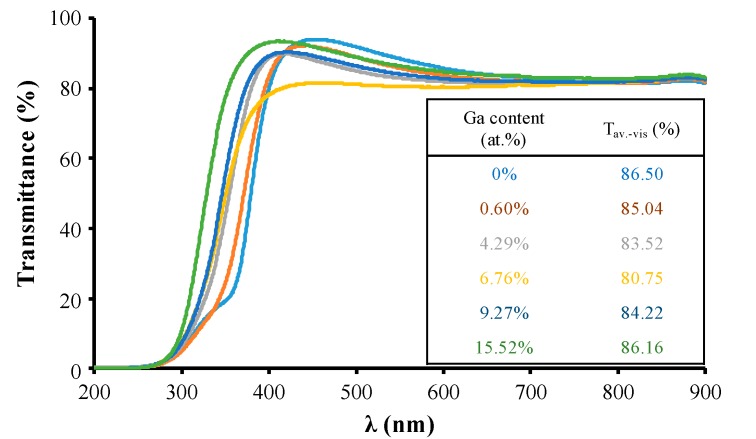
The variation in film transmittance as a function of Ga content in ZnO films.

The film’s transmittance is shown in Figure 6. Regardless of the Ga content of the ZnO, the films presented high transmittance in the visible region (400 nm to 800 nm). Their average transmittance was always above 80%. When the Ga content was increased from 0 at % to 6.76 at %, the number of defects, such as (Ga_Zn_) and (Ga*_i_*), and Ga clusters increased, and the light scattering by these defects and clusters was enhanced. Hence, a slight decrease in the film’s transmittance was observed in this region. With further increases in Ga content, gallium clusters gathered together, increasing in size while decreasing in number. The film’s transmittance then improved. In addition, with increases in Ga content, blue shifting of the absorption edge was observed, corresponding to the expansion of the film’s band gap. According to Vagard’s law mentioned in [26], because the band gap of Ga_2_O_3_ is about 4.9 eV larger than that of ZnO, the band gap of Zn_1-*x*_Ga*_x_*O films should increase linearly as the Ga doping amount increases.

**Figure 7:**
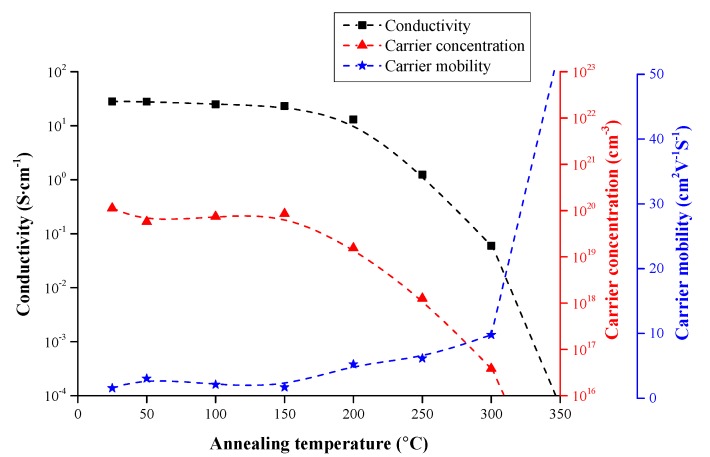
The electrical thermal stability of the ZnO film with 4.29 at % Ga under normal atmospheric conditions.

The film’s electrical thermal stability is shown in Figure 7. A ZnO film with 4.29 at % Ga was annealed at various temperatures under normal atmospheric conditions. When the annealing temperature was below 200 °C, the film’s conductivity was slightly degraded with increasing annealing temperature because the oxygen vacancies (*V*_O_) in the ZnO film were occupied by oxygen in oxygenated conditions. As reported by A.F. Kohan [27], oxygen vacancies, as the most important intrinsic donor defect, are favorable to the film’s n-type conductivity. Therefore, with the reduction in the number of oxygen vacancies, the carrier concentration also decreased. In addition, due to the reduction in the number of *V*_O_ defects, the carrier scattering by these defects was restricted, so the carrier mobility improved.

The melting point of gallium is only around 29.7 °C [28], and the partial pressure of indium atoms on the surface of the film is much lower than the saturated vapor pressure of indium. Therefore, when the annealing temperature exceeded 200 °C, the neutral gallium atoms that could not replace Zn cations obtained sufficient energy to migrate to the surface of the ZnO film, where they easily reacted with oxygen and formed insulating oxides. Hence, the carrier concentration and conductivity significantly dropped, and the carrier mobility increased. When the annealing temperature was higher than 300 °C, the film became completely insulating.

**Figure 8:**
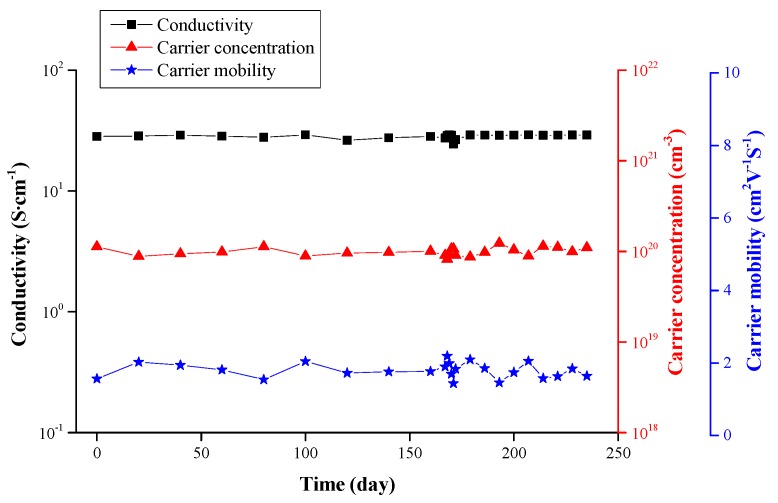
The electrical time stability of the ZnO film with 4.29 at % Ga at ambient temperature.

The film’s electrical time stability is given in Figure 8. The ZnO film with 4.29 at % Ga was placed in normal atmospheric conditions at ambient temperature for more than 240 days. With prolonged analysis time, the film’s electrical properties remained almost unchanged. In other words, the occupation of oxygen vacancies by oxygen atoms in oxygenated conditions could be activated only at high temperatures. Furthermore, gallium atoms cannot migrate in a ZnO lattice spontaneously at room temperature. Accordingly, the ZnO/Ga films presented satisfactory time stability.

## 3. Materials and Methods 

### 3.1. Deposition Parameters

Ga-doped ZnO (GZO) thin films with a thickness of about 100 nm were deposited on Corning 1737F glass substrates at room temperature via rf sputtering. The substrate holder rotation rate was about 10 rpm. Pure Ar was used as the working gas, and the working pressure was about 0.67 Pa. The sputtering power applied to the ZnO target (purity 99.95%, diameter 2.54 cm) was fixed at 80 W, while the sputtering power applied to the Ga_2_O_3_ target (purity 99.95%, diameter 2.54 cm) varied from 0 to 80 W. The power densities applied on ZnO and Ga_2_O_3_ targets are given in Table 1 and Table 2. Other sputtering parameters maintained during the deposition of GZO thin films are shown in Table 3.

### 3.2. Materials Characterizations

The thickness of the films was measured with a surface profilometer (Surfcoder SE-2300, Kosaka Laboratory Ltd., Tokyo, Japan). The composition of the films was detected with an electron probe X-ray microanalyzer (EPMA, JXA-8900R, JOEL, Tokyo, Japan). The phase structures of the films were analyzed with an X-ray diffractometer (XRD, X’Pert PRO MRD, Philips PANalytical, Almelo, The Netherlands) using the thin film mode with an incidence angle of 1°. Hall effect analysis (AHM-800B, Agilent Technologies, Santa Clara, CA, USA) with van der Pauw’s configuration was employed to investigate the electrical properties of the films. Finally, the transmittance of the films was measured with a UV-Vis spectrophotometer (JASCO-V650, JASCO, Tokyo, Japan).

## 4. Conclusions

GZO films were deposited via rf sputtering. The optoelectronic properties of the films as a function of Ga content were investigated. It was found that, when the gallium content was near 4.29 at %, ZnO/Ga films possess optimal electrical properties. All such ZnO films present high transmittance in the visible region, regardless of gallium content. In addition, due to the occupation of oxygen vacancies by oxygen atoms and the migration of gallium atoms in ZnO film, the film’s electrical properties are degraded under high temperatures in oxygenated conditions. However, this degradation was not observed in the time stability examination. This result suggests that ZnO/Ga films can withstand long-term loads at ambient temperatures but are not suitable for applications in high-temperature environments.

## Figures and Tables

**Table 1:** The power densities applied on ZnO target.

Power (W)	Power Density (W/cm^2^)
80	3.95

**Table 2:** The power densities applied on Ga_2_O_3_ target.

Power (W)	Power Density (W/cm^2^)
0	0
20	0.99
30	1.48
40	1.97
50	2.47
80	3.95

**Table 3:** Sputtering parameters maintained during the deposition of GZO thin films.

Parameter	Values	
Background pressure (Pa)	<6.7 × 10^−4^	
Working pressure (Pa)	0.67	
Films thickness (nm)	100	
Rotation speed (rpm)	10	
Substrate	Glass/Silicon	
Power (W)	ZnO	80
	Ga_2_O_3_	0–80
